# Pharmacokinetics of BMEDA after Intravenous Administration in Beagle Dogs

**DOI:** 10.3390/molecules19010538

**Published:** 2014-01-03

**Authors:** Chih-Hsien Chang, Si-Yen Liu, Te-Wei Lee

**Affiliations:** 1 Isotope Application Division, Institute of Nuclear Energy Research, Taoyuan 32546, Taiwan; E-Mails: chchang@iner.gov.tw (C.-H.C.); b9029506@yahoo.com.tw (S.-Y.L.); 2 Department of Biomedical Imaging and Radiological Sciences, National Yang-Ming University, Taipei 11221, Taiwan

**Keywords:** acute toxicity, radiopharmaceutical, BMEDA, ^188^Re-BMEDA-liposome, pharmacokinetics

## Abstract

The pharmacokinetics of *N*,*N*-bis(2-mercapatoethly)-*N*',*N*'-diethylenediamine (BMEDA), a molecule that can form a chelate with rhenium-188 (^188^Re) to produce the ^188^Re-BMEDA-liposomes, was studied. In this work, beagles received a single injection of BMEDA, at doses of 1, 2, or 5 mg/kg; the concentration of BMEDA in the beagles’ plasma was then analyzed and determined by liquid chromatography-mass spectrometry/mass spectrometry. Based on the pharmacokinetic parameters of BMEDA, we found that male and female animals shared similar patterns indicating that the pharmacokinetics of BMEDA is independent of gender differences. In addition, the pharmacokinetics of BMEDA was seen to be non-linear because the increase of mean AUC_0–t_ and AUC_0–∞_ values tend to be greater than dose proportional while the mean Vss and CL values of BMEDA appeared to be dose dependent. The information on the pharmacokinetics of BMEDA generated from this study will serve as a basis to design appropriate pharmacology and toxicology studies for future human use.

## 1. Introduction

Colorectal cancer is the third most common cancer and the fourth most common cause of cancer mortalities worldwide [1,2]. While various treatment regimens and cancer drugs are available for colorectal cancer, a variety of new treatments for this cancer are being developed [3,4], one of which includes the use of nanotechnology [5]. Nanoliposomes, a double-membrane lipid vesicle, are one of the most popular drug delivery systems that has been studied for the controlled release of tumor-related chemotherapeutic agents [6]. Dosimetry measurement studies have shown that liposomes themselves can deliver therapeutic radionuclides to tumors, thus exposing them to high levels of radiation, while sparing bone marrow and lowering radiation levels in the liver and spleen [7,8,9,10].

Rhenium-188 (^188^Re) is an ideal radionuclide for therapeutic use because of its maximum beta emission of 2.12 MeV and 155 keV gamma emission used for therapeutics and for imaging, respectively. In addition, it has a short physical half-life of 16.9 h [11]. The ^188^Re-*N*,*N*-bis-(2-mercaptoethyl)-*N*',*N*'-diethylethylenediamine (BMEDA)-liposome is a radioactive nanoparticle developed for the diagnosis and therapy of tumors, with proven efficacy for experimental cancer therapy in murine colon carcinoma ascites [9,12], lung metastasis [13], and solid tumor mouse models [14,15]. Further clinical applications of ^188^Re-BMEDA-liposome would first require an evaluation of its safety through animal models.

Acute radiotoxicity studies in animals are required before therapeutic radiopharmaceuticals can be intended for human use. The information obtained from the study is useful for selecting dose in subsequent dose toxicity studies, providing preliminary identification of target organs to which toxicity being found and revealing delayed toxicity. To evaluate therapeutic strategies of ^188^Re-liposomes for clinical use, extended acute toxicity studies of ^188^Re-liposomes have been performed on rats [16]. BMEDA is a small molecule and used as a chelator with ^188^Re, and ^188^Re-BMEDA is further encapsulated in the liposome. For an Investigational New Drug application, the toxicity of BMEDA is also needed to prove the safety of this chelator.

Pharmacokinetics is defined as the generation of pharmacokinetic data to assess systemic exposure of a new therapeutic agent, focusing on the rate that chemicals enter the body. The purpose of this study is to evaluate the pharmacokinetics of BMEDA in beagle dogs via single intravenous injection administration. The information generated from the pharmacokinetic study will serve as a basis to design appropriate pharmacology and toxicology studies for future human use.

## 2. Results

### Method Validation

Selectivity was confirmed through the chromatograms of blank samples and blank samples spiked with BMEDA. Under the given conditions, BMEDA was eluted at a retention time of 1.8 min for the plasma samples, and there was no interference at the same retention time. Calibration curves for BMEDA were shown to have linear regression in the concentration range of 10 to 3,000 ng/mL for plasma; the coefficient of determination (*r*^2^) was greater than 0.995 for all curves, indicating good linear regression in the studied concentration range. Table 1 reveals that intra- and inter-day precisions (RSD %) were less than 10.7%, and accuracies (RE %) for intra- and inter-day assays were less than 12.3%. The lower limit of quantification (LLOQ) was 10 ng/mL for BMEDA.

**Table 1:** Intra-day and Inter-day Precision and Accuracy of BMEDA in K_2_ EDTA Dog Plasma.

Nominal concentration (ng/mL)	Observed concentration (ng/mL)	Precision (RSD %)	Accuracy (% Bias)
*Intra-assay (n = 6)*			
10	8.7 ± 0.6	6.9	−12.9
30	29.4 ± 1.6	5.5	−2.1
240	269.5 ± 14.0	5.2	12.3
2400	2303.4 ± 199.6	8.7	−4.0
*Inter-assay (n = 18)*			
10	9.5 ± 1.0	10.7	−4.8
30	30.2 ± 1.6	5.4	0.7
240	257.0 ± 16.5	6.4	7.1
2400	2229.9 ± 142	6.4	−7.1

Observed mass concentrations are expressed as mean ± SD.

The matrix effect and recovery for determination of BMEDA is summarized in Table 2 and these results show that the detection and extraction methods for these samples are reliable and acceptable.

**Table 2:** Matrix effect and recovery of BMEDA in K_2_ EDTA Dog Plasma.

Conc. (ng/mL)	Matrix effect (%)	Recovery (%)
30	104.3	98.9
240	110.0	86.3
2400	107.4	89.3
Average	107.2 ± 2.9	91.5 ± 6.6

Data are expressed as mean ± standard deviation.

**Figure 1:**
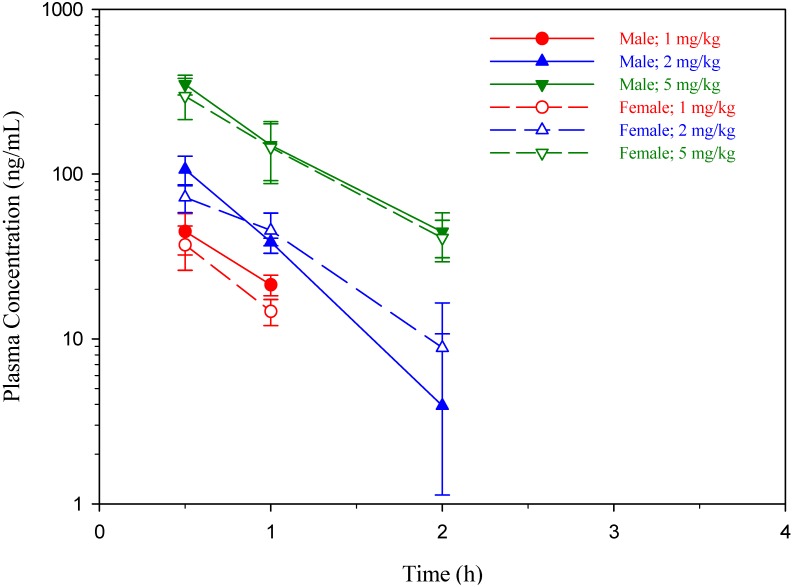
Mean Plasma Concentration-Time Profiles of BMEDA in Beagle Dogs after Intravenous Injection of BMEDA at 1, 2 and 5 mg/kg. Data are expressed as mean ± standard deviation.

The dogs in the test groups were injected with BMEDA at either 1, 2 or 5 mg/kg followed by analysis of BMEDA concentration in dog plasma. The plots of mean plasma concentration of BMEDA in dogs are depicted in Figure 1. In all plasma samples collected at five consecutive time points post injection of BMEDA, we found that BMEDA detection in plasma was possible for samples in response to 1 and 2 mg/kg collected at 1 and 2 h post-BMEDA injection and samples in response to 5 mg/kg collected at 0.5, 1.0, and 2.0 h post-BMEDA injection. Analyzing the plasma concentration-time profiles of BMEDA in dogs across all three treatment regimens showed that both sexes shared a similar pattern. A summary of the pharmacokinetic parameters for BMEDA in beagle dogs is shown in Table 3 and Table 4.

The C_max_ of BMEDA for the given dosages (1, 2, and 5 mg/kg) in male dogs averaged to 44.9, 107 and 350 ng/mL. When compared between the three groups, the C_max_ in the 2 mg/kg group was 2.38 times higher than that of the 1 mg/kg group, while the 5 mg/kg group was 7.80 times greater than that of the 1 mg/kg one. Likewise, the C_max_ of BMEDA in female dogs, at the same dosages, was 37.2, 73.2 and 297 ng/mL; compared to the C_max_ in the 1 mg/kg group, the value in the 2 mg/kg group was 1.97 times higher while that of the 5 mg/kg group was 7.98 times higher. These results indicate that the mean C_max_ values of BMEDA increased with dose, independent of gender, across the range of 1–5 mg/kg and increased dose-proportionally up to 2 mg/kg.

The mean AUC_0–t_ values of BMEDA for the given dosages (1, 2, and 5 mg/kg) in male dogs were 26.6, 71.7 and 310 h·ng/mL. The value of the 2 mg/kg group and 5 mg/kg group was 2.70 times and 11.7 times higher than that of the 1 mg/kg group, respectively. In addition, the mean AUC_0–t_ values of BMEDA for the same given dosages in female dogs were 22.3, 67.7 and 278 h·ng/mL. When compared to the mean AUC_0–t_ value in the 1 mg/kg group, the value of the 2 mg/kg group was 3.04 times higher, and 12.5 times higher for the value of the 5 mg/kg group, than that of the 1 mg/kg group.

The mean AUC_0–∞_ values of BMEDA for the given dosages (2 and 5 mg/kg) in male dogs were 96.8 and 343 h·ng/mL, and a comparison revealed that the value in 5 mg/kg group was 3.54 times higher than that of the 2 mg/kg group. Meanwhile, the mean AUC_0-∞_ values of BMEDA for the same given dosages in female dogs were 83.1 and 310 h·ng/mL, and compared to the mean AUC_0-∞_ value in the 2 mg/kg group, the value in the 5 mg/kg group was 3.73 times higher. These results indicate that, regardless of gender, the mean AUC_0–t_ and AUC_0–∞_ values of BMEDA increased more than proportionally to the dose increase.

**Table 3:** Pharmacokinetic Parameters of BMEDA in Beagle Dogs after Intravenous Injection of BMEDA at 1, 2 and 5 mg/kg.

Parameter	Dose (mg/kg)	Male (N = 3)		Female (N = 3)		Combined (N = 6)	
		Mean	SD	Mean	SD	Mean	SD
C_max_ (ng/mL)	1	44.9	12.7	37.2	11.1	41.1	11.5
	2	107	21.6	73.2	12.4	89.9	24.2
	5	350	48.3	297	83.4	324	67.4
AUC_0–t_ (h·ng/mL)	1	26.6	8.04	22.3	6.14	24.5	6.83
	2	71.7	18.4	67.7	15.6	69.7	15.4
	5	310	62.2	278	87.4	294	70.0
AUC_0–∞_ (h·ng/mL)	1	ND	ND	ND	ND	ND	ND
	2	96.8 ^a^	NC ^a^	83.1 ^a^	NC ^a^	90.0 ^b^	9.69 ^b^
	5	343	75.2	310	95.0	326	78.8
Vss (mL/kg)	1	ND	ND	ND	ND	ND	ND
	2	18,887 ^a^	NC ^a^	25,688 ^a^	NC ^a^	22,288 ^b^	4809 ^b^
	5	14,473	2284	17,243	5281	15,858	3942
CL (mL/h/kg)	1	ND	ND	ND	ND	ND	ND
	2	20,663 ^a^	NC ^a^	24,077 ^a^	NC ^a^	22,370 ^b^	2414 ^b^
	5	15,074	3475	17,126	4777	16,100	3901
T_1/2_ (h)	1	ND	ND	ND	ND	ND	ND
	2	0.5 ^a^	NC ^a^	0.6 ^a^	NC ^a^	0.6 ^b^	0.1 ^b^
	5	0.5	0.1	0.5	0.1	0.5	0.1
MRT_0–∞_ (h)	1	ND	ND	ND	ND	ND	ND
	2	0.9 ^a^	NC ^a^	1.1 ^a^	NC ^a^	1.0 ^b^	0.1 ^b^
	5	1.0	0.1	1.0	0.0	1.0	0.1

ND: Not determined due to insufficient data points in the elimination phase for λz determination; NC: Not calculated; ^a^ N = 1, because there were insufficient data points in the elimination phase for λz determination in two male and two female Beagle dog at 2 mg/kg dose level; ^b^ N = 2.

The mean Vss values of BMEDA for the given dosages (2 and 5 mg/kg) in male dogs were 18,887 and 14,473 mL/kg, while in female dogs, they were 25,688 and 17,243 mL/kg. The Mean CL values of BMEDA for the given dosages (2 and 5 mg/kg) in male dogs were 20,663 and 15,074 mL/h/kg and 24,077 and 17,126 mL/h/kg in female dogs. This data indicates that the mean Vss and CL values appeared to be dose-dependent from 2 to 5 mg/kg.

The mean T_1/2_ values of BMEDA for the given dosages (2 and 5 mg/kg) in male dogs were 0.5 and 0.5 h, and 0.6 and 0.5 h in female dogs. The mean MRT_0–∞_ values of BMEDA for the given dosages (2 and 5 mg/kg) in male dogs were 0.9 and 1.0 h and 1.1 and 1.0 h in female dogs. These results indicate that the mean T_1/2_ and MRT_0–∞_ values appear to be independent of dose from 2 to 5 mg/kg.

## 3. Discussion

During the stages of pharmaceutical development, from discovery to clinical study trials, the examination of the pharmacokinetics of a chemical substance is the utmost importance to obtain information on its absorption, distribution, metabolism and excretion, to aid in relating concentration to potential toxicity, and to aid in the understanding of the mechanisms by which test substances use to produce toxicity in the body.

**Table 4:** Ratio of Mean AUC_0–t,_ AUC_0–∞_ and C_max_ of BMEDA Compared to 1 mg/kg Dose Level of BMEDA in Beagle Dogs after Intravenous Injection of BMEDA at 1, 2 and 5 mg/kg.

Parameter	Dose (mg/kg)	Male (N = 3)		Female (N = 3)		Combined (N = 6)	
		Mean	Ratio	Mean	Ratio	Mean	Ratio
C_max_ (ng/mL)	1	44.9	−	37.2	−	41.1	−
	2	107	2.38	73.2	1.97	89.9	2.19
	5	350	7.80	297	7.98	324	7.88
AUC_0-t_ (h·ng/mL)	1	26.6	−	22.3	−	24.5	−
	2	71.7	2.70	67.7	3.04	69.7	2.84
	5	310	11.7	278	12.5	294	12.0
AUC_0-∞_ (h·ng/mL)	1	ND	−	ND	−	ND	−
	2	96.8 ^a^	−	83.1 ^a^	−	90.0 ^b^	−
	5	343	3.54 ^c^	310	3.73 ^c^	326	3.62 ^c^

−: Not available. ND: Not determined due to insufficient data points in the elimination phase for λz determination; ^a^ N = 1, because there were insufficient data points in the elimination phase for λz determination in two male and two female Beagle dog at 2 mg/kg dose level; ^b^ N = 2; ^c^ Compared to 2 mg/kg dose level of BMEDA.

The most convenient method of preparing radioactive liposomes is to use a conjugate system from which the ^188^Re-BMEDA-liposome was created, having both a chelator (BMEDA) and rhenium-188 on the inside of the liposome. At the beginning of manufacturing, rhenium-188 was chelated with BMEDA, which in turn formed the ^188^Re-BMEDA SNS/S complex; depending on the lipophilic characterization of BMEDA, the complex could cross the lipid bilayer and enter the inner space of the liposome. Once inside, the amine group of the complex would gain protons due to the ammonium sulfate gradient, and as a result, the complex becomes hydrophilic, further entrapping itself in the interior of the liposome [17,18,19].

From experiment cancer therapy studies, the ^188^Re-BMEDA-liposome showed a therapeutically beneficial effect [12,13,14,15,20]. However, prior to the Investigational New Drug application, where an estimate of drug’s safety for initial human use should be performed, both animal pharmacology and toxicology studies for both the ^188^Re-BMEDA-liposome as well as the BMEDA must be performed, since BMEDA is a particular ingredient in the ^188^Re-BMEDA-liposome. Within a 14-day period, the extended acute toxicity of BMEDA had been evaluated in ICR mice where the No Observed Adverse Effect Level (NOAEL) of BMEDA for them was 3 mg/kg [21]. Likewise, the NOAEL of BMEDA in Beagle dogs was 1 mg/kg [22]. Overall, the relationship between the systemic exposure of BMDEA in beagle dogs and its pharmacokinetics was established. Through this, we can gain insight into the associations between the potential toxicity of BMEDA and the dose range for BMEDA in dogs, which may be compliant with linear or non-linear kinetics.

The basic pharmacokinetic parameters of drugs can be used to establish whether clearance is saturated in a dose-dependent fashion; the proportionality of dose range of drugs to its AUC is used as a main criterion to determine the kind of kinetics exhibited by drugs [23]. However, in this study, even though the area under the plasma concentration-time curve (AUC) seemed to be proportional to increase in BMEDA concentrations, the rise in AUC was not directly proportional to the dose range of BMEDA; rather, it was greater than the proportional dose (Table 4). In addition, the mean Vss and CL values that were not said to be dose-independent ranged from 2 mg/kg to 5 mg/kg; a decrease in elimination can be a partial explanation to the disproportionate values of AUC due to the AUC was a given dose divided by the CL value [23]. The different doses of BMEDA did not result in the parallel plasma concentration-time course as a result of the initial decline of BMEDA plasma concentrations in the highest dose group was slower than that in the other dose groups (Figure 1). This indicates that the rate of elimination was not directly proportional to the plasma concentration of BMEDA. In this preliminary results, the pharmacokinetic study of BMEDA might reveal a demonstration of non-linear kinetics; several factors contributing to it include: a decrease in elimination [24,25], the capacity-limited hepatic metabolism [26], and the saturation of the binding of BMEDA to plasma or tissue proteins [27,28].

Typically, when the binding of a chemical substance to plasma protein is concentration-dependent and site-specific, once the specific binding sites are saturated, the volume of distribution (Vss) should increase [23,29,30]. In contrast, the Vss for BMEDA in this study decreased, and this phenomenon may relate to the non-specific binding of BMEDA to macromolecules present in either the tissues or bloodstream, of which two types can occur simultaneously. Since the volume of distribution is directly and inversely related to the binding of BMEDA for both plasma and tissue proteins, gains in one area offset losses in another [23]. Considering the capacity-limited hepatic metabolism as a result of exposure to a high dose of test substance, a decrease in total body clearance would be found [26,31]; consistent with this is decrease of CL for BMEDA in the highest concentration.

In this study, the pharmacokinetics of BMEDA was established, and even though the elimination of most test substances followed linear kinetics, others followed non-linear kinetics. This behavior had been found in several studies similar to BMEDA, not only limited to exposure in high concentration of BMEDA.

## 4. Experimental

### 4.1. Reagents

*N*,*N*-Bis(2-mercapatoethly)-*N*',*N*'-diethylenediamine (BMEDA) was purchased from ABX GmbH (Radeberg, Germany) and the chemical structures were verified with ^1^H- and ^13^C-NMR. Betaxolol hydrochloride was obtained from Sigma-Aldrich (St. Louis, MO, USA).

### 4.2. Experimental Animals

Beagle dogs were obtained from Marshall BioResources (North Rose, NY, USA). Beagles were 9 months of age, weighing between 7.4 to 10.3 kg for males and between 5.5 to 7.7 kg for females. The experimental protocol and animal husbandry was approved by the Institutional Animal Care and Use Committee at QPS Taiwan, which had earned the Association for Assessment and Accreditation of Laboratory Animal Care accreditation. Prior to pharmacokinetic investigations of BMEDA, beagle dogs received a series of examinations to ensure animal health.

There were 24 beagle dogs in total (12 males and 12 females) used for this pharmacokinetic study in which dogs were randomly allocated into control group and three test group (three males and three females for each group). The dogs in the test groups were injected intravenously with BMEDA, and each group correlates with a different dosage regimen consisting of either 1 mg/kg, 2 mg/kg, or 5 mg/kg; dogs in the control group, consisting of six animals with an equal number of males and females, were injected with isotonic sodium chloride solution. Blood collection was performed at time points before dosing, as well as at 0.5, 1, 2, 6 and 24 h post-BMEDA dosing. Blood samples (~1 mL) from beagle dogs were collected from the cephalic vein (non-injection site) and transferred into K_2_EDTA anticoagulant tubes. Blood was kept on ice until centrifugation was performed within one hour after blood collection (1000 ×*g* and 4 °C for 15 min). All plasma samples were stored at −20 °C before analysis.

### 4.3. Liquid Chromatography-Mass Spectrometry/Mass Spectrometry

All analysis was performed with the API 4000TM LC/MS/MS Triple Quadrupole System (AB SCIEX, Concord, ON, Canada). All plasma samples were added with betaxolol hydrochloride, which was used as the internal standard; proteins were precipitated from samples with acetonitrile and hydrochloric acid with a volume to volume ratio of 100:0.1. The samples were analyzed by normal-phase high-performance liquid chromatography with atmospheric pressure chemical ionization MS/MS detection in which positive (M+H)^+^ ions for BMEDA and betaxolol were monitored with MRM mode. The multiple-reaction monitoring transitions were *m/z* 237.2 to *m/z* 100.2 for BMEDA and *m/z* 308.2 to *m/z* 116.1 for betaxolol. A linear calibration curve using 1/x^2^ weighted least-squares regression analysis was created by the ratio of analyte-to-internal standard peak area for the calibration standards. Ranges of BMEDA concentrations were from 10 to 3,000 ng/mL; a minimum of seventy-five percent of the calibration standards was needed to be within calibration range was needed while the lower limit of quantification for this assay was 10 ng/mL. The selectivity of this assay was evaluated by individually analyzing six batches of dog plasma neither BMEDA nor the internal standard therein. No interference peak area in the peak of the retention time of either BMEDA or internal standard, and in the mean analyte peak area was the minimum needed. The matrix effects on BMEDA and the internal standards of dog plasma were evaluated by comparing the mean peak areas obtained from the BMEDA spikes into extracts of blank matrix to the mean peak areas obtained from the same solutions used in preparing test samples.

### 4.4. Method Validation

Calibration curves were established by using blank samples of plasma spiked with different amounts of BMEDA. Stock solution diluted with methanol was used to form a series of concentrations from 10 to 3,000 ng/mL. The concentration–response relationship from the present method indicates linearity over a concentration range of 10–3,000 ng/mL with a coefficient of estimation (*r*^2^) of at least 0.999. The quality control concentration for intra-assay and inter-assay variability was determined by quantitating six and eighteen replications at concentrations of 10, 30, 240 and 2,400 ng/mL on the same day and consecutive days, respectively. The accuracy (bias %) was calculated from the mean value of observed concentration (C_obs_) and the nominal concentration (C_nom_) as follows: accuracy (%) = [(C_obs_ − C_nom_)/C_nom_] × 100. The relative standard deviation (R.S.D.) was calculated from the observed concentrations as follows: precision (%) = [standard deviation (S.D.)/C_obs_] × 100. Accuracy and precision values of the actual range of experiment concentrations were within ±20% and considered acceptable. Matrix effect tests were used to evaluate the suppression or enhancement of the analyte and the internal standard response by the matrix. The matrix effects were determined in K_2_ EDTA dog plasma at three concentrations (30, 240, and 2,400 ng/mL, *n* = 5) for BMEDA. Matrix effect was evaluated by comparing the mean peak areas obtained from the analyte spiked into extracts of blank matrix (“unextracted samples”, representing 100% recovery) to those obtained from solutions with the same nominal concentration prepared in the same type and amount of solvent (“neat samples”). The neat samples served as a reference, and the matrix effect was calculated as follows: Matrix Effect (%) = (Mean Peak Area of Un-extracted Samples/Mean Peak Area of Neat Samples) × 100. The %CV of the results for the three concentrations tested could not exceed 20.0% in order to be considered consistent across the validated assay range. The purpose of the recovery test was to evaluate the efficiency of the protein precipitation extraction process. Recovery was determined at three concentrations (30, 240, and 2,400 ng/mL, *n* = 5) for BMEDA. The recovery of the analyte in this assay was evaluated by comparing the mean peak areas from the analyte added to and recovered from the biological matrix (extracted samples) to the peak areas from the un-extracted samples as described for the matrix effect test. Recovery was calculated as follows: Recovery (%) = (Mean Peak Area of Eextracted Samples/Mean Peak Area of Unextracted Samples) × 100. The %CV of the results for the three concentrations tested could not exceed 20.0%.

### 4.5. Pharmacokinetic Analyses

The pharmacokinetic parameters included half-life (t_1/2_), maximum concentration (C_max_), area under the concentration-versus time (AUC), mean residence time (MRT), total body clearance (CL), and an estimate of the volume of distribution at steady-state (Vss); these were determined by standard model independent methods based on the plasma concentration-time data [32]. All the data are presented in mean ± standard deviation. The pharmacokinetic analyses were performed using WinNonlin^®^ version 5.2.1 (Pharsight Corporation, Mountain View, CA, USA). All concentration values below quantifiable limit (BQL) before the first quantifiable concentration and after the last quantifiable concentration were treated as zero for all pharmacokinetic and statistical evaluation.

## 5. Conclusions

Using the API 4000TM LC/MS/MS Triple Quadrupole System, the BMEDA concentrations in the plasma of beagle dogs was revealed. Based upon the retrieval of BMDEA from dogs’ plasma where an increase in BMEDA dose did not increase proportionally to the mean C*_max_* values of BMEDA and the pharmacokinetic parameters of BMEDA, BMEDA followed non-linear kinetics at exposure to high concentration (5 mg/kg). This provides a point of reference for the considerations of pharmacology and toxicology studies of BMEDA in the human treatment plans.
